# Decomposing motion that changes over time into task-relevant and task-irrelevant components in a data-driven manner: application to motor adaptation in whole-body movements

**DOI:** 10.1038/s41598-019-43558-z

**Published:** 2019-05-10

**Authors:** Daisuke Furuki, Ken Takiyama

**Affiliations:** Department of Electrical and Electronic Engineering, Tokyo University of Agriculture and Technology, Koganei-shi, Tokyo, 184-8588 Japan

**Keywords:** Motor control, Sensorimotor processing

## Abstract

Motor variability is inevitable in human body movements and has been addressed from various perspectives in motor neuroscience and biomechanics: it may originate from variability in neural activities, or it may reflect a large number of degrees of freedom inherent in our body movements. How to evaluate motor variability is thus a fundamental question. Previous methods have quantified (at least) two striking features of motor variability: smaller variability in the task-relevant dimension than in the task-irrelevant dimension and a low-dimensional structure often referred to as synergy or principal components. However, the previous methods cannot be used to quantify these features simultaneously and are applicable only under certain limited conditions (e.g., one method does not consider how the motion changes over time, and another does not consider how each motion is relevant to performance). Here, we propose a flexible and straightforward machine learning technique for quantifying task-relevant variability, task-irrelevant variability, and the relevance of each principal component to task performance while considering how the motion changes over time and its relevance to task performance in a data-driven manner. Our method reveals the following novel property: in motor adaptation, the modulation of these different aspects of motor variability differs depending on the perturbation schedule.

## Introduction

In our daily lives, we repeatedly perform various desired movements, such as grasping a cup, throwing a ball, and playing the piano. To achieve the desired movements, the human motor system needs to resolve at least two difficulties inherent in our body motion^1^. One difficulty is movement variability. Due to the variability inherent in various stages, such as the acquisition of sensory information^2^, the neural activity that occurs during motor planning^3^, and the muscle activities that occur during motor execution^4^, even sophisticated athletes and musicians cannot precisely repeat the same exact movements. However, our motor systems somehow tame these variabilities to achieve the desired movements^5^. The second difficulty is the large number of degrees of freedom (DoFs) inherent in the human motor system^1,6^. The numbers of joints, muscles, and neurons in this system exceed those necessary to achieve the desired movements, resulting in an infinite number of possible joint configurations, muscle activities, and neural activities that can correspond to a desired movement^7–10^. Our motor systems somehow resolve these difficulties (i.e., variability and the large number of DoFs) to generate the desired movements.

Although it remains unclear how this taming of movement variability is achieved, one possible answer lies in the decomposition of motor variability into task-relevant and task-irrelevant variabilities. We compensate for the portion of motor variability that is relevant to achieving our desired movements (i.e., task-relevant variability)^11–15^, while we do not significantly compensate for the portion that is irrelevant to achieving the desired movements (i.e., task-irrelevant variability). The compensation of task-relevant variability can be observed in movement kinematics^11–14^, muscle activities^16,17^, and neural activities^15^. This striking feature of our motor variability enables the achievement of the desired movements despite the presence of movement variability.

Techniques for evaluating task-relevant and task-irrelevant variabilities have been developed in several studies. In the uncontrolled manifold (UCM) approach, task-relevant and task-irrelevant variabilities are evaluated mainly in terms of joint angles and angular velocities. This method focuses on the kinematic parameters relevant to task achievement, such as the hip joint position in stand-and-sit motions^11^ or the hand position in arm-reaching movements^18^. The Jacobian matrix, which contains the derivatives of those kinematic parameters concerning joint angles or angular velocities, enables the definition of the null space around the joint angles or angular velocities averaged across trials. The variability throughout this null space can be defined as the task-irrelevant variability. Most previous studies have revealed that the task-relevant variability of joint angles and angular velocities is less than the task-irrelevant variability. Notably, the UCM approach focuses on forward kinematics, mapping joint angles and angular velocities to joint positions and velocities in external coordinates. In contrast, the tolerance, noise, and covariation (TNC) analysis approach^13^ and the goal-equivalent manifold (GEM) analysis approach^14^ focus on task functions that define the relationships between kinematic parameters and task performance. For example, a thrown dart or ball can be modeled as a parabola. Let the release position and velocity on the vertical axis be denoted by *p* and *v*, respectively; then, the maximum height of the released dart or ball can be expressed as $h=p+\frac{{v}^{2}}{2g}$, where *g* is the gravitational acceleration. When the task is to control *h*, the relation among *h*, *p*, and *v* is the task function. For example, with a small value of *d* (i.e., ${d}^{2}\simeq 0$), the corresponding slight changes in the release position, $p+\frac{v}{g}d$, and the release velocity, $v-d$, together cause no change in *h*. In other words, the changes in *p* and *v* caused by *d* cancel each other out, resulting in the same height *h*; thus, the variability represented by these slight changes can be regarded as the task-irrelevant variability. In the TNC and GEM methods, the task-relevant and task-irrelevant variabilities are evaluated based on such task functions.

These techniques have certain advantages and disadvantages (e.g., several disadvantages of the UCM method were noted by Müller & Dagmar^13^). The UCM method enables the evaluation of motion that changes over time, but it does not consider task functions. Consequently, this framework is not always suitable in situations in which the kinematic parameters are nonlinearly relevant to the task achievements, as in the case of the quadratic function of *v* in the parabola mentioned above. Because forward kinematics involves nonlinear functions of the joint angles and angular velocities, the UCM method requires local linear approximations around representative joint angles or angular velocities based on the Jacobian matrix. Due to these linear approximations, the UCM model assumes the kinematic variability that is averaged across all trials. These approximations result in difficulty in simultaneously considering the changes in motor variability as the average kinematics change, such as the changes that occur before, during, and after motor learning (although it is possible to discuss those situations separately^18^). The GEM approach also considers local linear approximations of the nonlinear task function; thus, it also considers the variability in the task parameters averaged across all trials. Although the GEM method can consider task functions, it has difficulty considering how motion changes over time in certain cases. For example, to consider how the motion changes over time in the example mentioned above using the GEM framework, it would be necessary to define how the position and velocity of the dart or ball 100 milliseconds before release are related to the maximum height. By contrast, the TNC approach enables the simultaneous consideration of the motor variability before, during, and after motor learning because this framework captures the whole variability in a nonparametric manner without local linear approximations; however, the TNC method is not always suitable for considering motion that changes over time for reasons similar to those for the GEM method, namely, it requires an explicit definition of the task function. Overall, each method has its own advantages and disadvantages; thus, no single existing framework can be used to simultaneously evaluate task-relevant and task-irrelevant variabilities when the average kinematics or task parameters are changing while considering both how the motion changes over time and the task function.

Motor variability also has another striking feature: it is embedded in a low-dimensional space that is referred to as synergy^6,19–22^. It has been suggested that to overcome the large number of available DoFs, the human motor system limits control of those DoFs to only those in this low-dimensional space. The concept of synergy has been (mainly) discussed in reference to kinematic data^20,22–24^ and electromyographic (EMG) data^19,21^. For both kinematic and EMG data, a low-dimensional space that can capture a high proportion of the motor variability has been found. Several methods have been developed for extracting this synergy, such as principal component analysis (PCA)^20,22^, nonnegative matrix factorization^21,25^ and the spatial-temporal decomposition of EMG data^19^.

Motor variability thus exhibits at least two characteristics: compensation of the task-relevant variability and a low-dimensional structure. However, most techniques for investigating motor variability address only one of these aspects. It is difficult to detect the low-dimensional structure of motor variability using methods for evaluating task-relevant and task-irrelevant variabilities. Similarly, it is difficult to evaluate task-relevant and task-irrelevant variabilities using techniques for extracting the low-dimensional structures of motor variability. Thus, the 1st principal component, the dimension that can explain the most significant portion of the variability among all dimensions, is not always either the most or least relevant to task performance. Although the UCM approach has been used for synergy extraction^8^, its primary advantage lies not in the extraction of the low-dimensional structure but rather in the evaluation of the task-relevant and task-irrelevant variabilities. Although a few studies have focused on linear discrimination analysis (LDA) for investigating the task-relevant low-dimensional space^26,27^, LDA enables only discrimination, e.g., distinguishing between the success or failure of a movement^28^. In contrast, the TNC and GEM methods allow motor performance to be characterized in terms of a continuous performance value. In summary, few methods enable the simultaneous quantification of the two most striking features of movement variability.

Here, we propose a flexible and straightforward machine learning framework for evaluating movement variability that combines the advantages of the various previous techniques: our framework can not only be used to evaluate task-relevant and task-irrelevant variabilities even when the average kinematics or task parameters are changing (e.g., before, during, and after motor learning) while considering how motion changes over time and the task function but also can reveal how each synergy is relevant to task performance by means of an extension of PCA. The current study relied on ridge regression^29^, a linear regression technique that is robust in the presence of measurement noise, has a definite relation to PCA, and can be used to evaluate how the motion of each part of the body at each moment in time is relevant to task performance in a data-driven manner^30^. Our technique thus enables the identification of task functions in a data-driven manner without requiring any explicit function, such as a parabola, or forward kinematics. First, we formalize the decomposition of motion data into task-relevant and task-irrelevant components by extending the ridge regression procedure. Second, we construct a novel experimental paradigm to investigate the relation of time-varying motion to task performance based on goal-directed and whole-body movements. We further discuss motor adaptation in the current experimental setting. Third, we validate the decomposition of motion data into task-relevant and task-irrelevant components based on our experimental data. Fourth, we clarify the relation between ridge regression and PCA, a popular method of extracting the low-dimensional space describing motor variability. In particular, we analytically reveal how each principal component is related to performance in the context of ridge regression. We also validate the analytical calculations based on our experimental data. Finally, we apply our method to motion data from whole-body and goal-directed movements before and after motor adaptation. Because our method enables us to discuss the modulation of movement variability before and after motor adaptation, we discuss the dependence of this modulation on the perturbation schedule.

## Results

Our program code can be downloaded from our website.

### Linear regression

The current study relied on linear regression to determine the relationship between motion data ${\boldsymbol{X}}\in {{\rm{R}}}^{T\times D}$ (in the form of a temporal sequence of joint angles and angular velocities) and performance data ${\boldsymbol{d}}\in {{\rm{R}}}^{T\times 1}$ based on the expression ${\boldsymbol{h}}={\boldsymbol{Xw}}$, where *T* and *D* denote the number of trials and the number of variables in the motion data, respectively; ${\boldsymbol{h}}\in {{\rm{R}}}^{T\times 1}$ is the predicted performance; and ${\boldsymbol{w}}\in {{\rm{R}}}^{D\times 1}$ is the best set of linear coefficients for predicting the performance^30^. ***X***_*t*_, representing the *t*th row of ***X*** or the motion data from the *t*th trial, consists of vectorized motion data, e.g., the data obtained by measuring the joint angles of the knee (${{\boldsymbol{q}}}_{k,t}\in {{\rm{R}}}^{1\times F}$) and hip (${{\boldsymbol{q}}}_{h,t}\in {{\rm{R}}}^{1\times F}$) for *F* time frames in the *t*th trial: ${{\boldsymbol{X}}}_{t}=({{\boldsymbol{q}}}_{k,t},{{\boldsymbol{q}}}_{h,t})\in {{\rm{R}}}^{1\times 2F}$.

### Ridge regression

We relied on a ridge regression procedure, which is robust against observation noise and is applicable to data that exhibit multicollinearity. The ridge regression enabled us to determine the best one-dimensional linear space ${\boldsymbol{w}}\in {{\rm{R}}}^{D\times 1}$ for predicting the output data ***d*** from the input data ***X*** by minimizing the following cost function:

1 $$E=\frac{1}{2}{({\boldsymbol{d}}-{\boldsymbol{Xw}})}^{T}({\boldsymbol{d}}-{\boldsymbol{Xw}})+\frac{\lambda }{2}{{\boldsymbol{w}}}^{T}{\boldsymbol{w}}.$$

Here, the first term on the right-hand side represents the fitting error, and the second term represents the regularization of ***w***, where *λ* is the regularization parameter. In the current study, *λ* was chosen to minimize the prediction error based on 10-fold cross-validation, allowing us to avoid overfitting^28^. Overfitting, which can arise in the absence of any regularization, will lead to a model that is more complicated than the true model.

Minimization of the cost function with respect to ***w*** leads to the optimal value of ***w***:

2 $${{\boldsymbol{w}}}^{\ast }={({\boldsymbol{X}}{{\boldsymbol{X}}}^{T}+\lambda {\boldsymbol{I}})}^{-1}{{\boldsymbol{X}}}^{T}{\boldsymbol{d}},$$

where ***I*** is the identity matrix. When ***XX***^*T*^ exhibits multicollinearity, it is difficult to calculate the inverse of ***XX***^*T*^ because of the rank deficiency. Adding the identity matrix scaled by the regularization parameter *λ* enables the calculation of the inverse of ***XX***^*T*^ + *λ****I*** and allows the output data to be predicted with a certain accuracy. Furthermore, the regularization parameter *λ* allows the appropriate ***w*** to be found even in the presence of observation noise because minimizing the cost function for ridge regression (equation (1)) is analytically equivalent to finding the appropriate ***w*** when nonzero observation noise exists (see the *Materials and Methods* section for the detailed mathematical calculations). The ridge regression procedure thus allows the relation between the motion data and performance data to be found while overcoming the multicollinearity inherent in the motion data in a manner that is robust against observation noise.

### Decomposition into task-relevant and task-irrelevant components

Once the best linear coefficients ***w*** have been estimated based on the measured performance data ***d*** and motion data ***X***, the estimated coefficients ***w*** enable not only performance prediction but also the decomposition of the motion data into a task-relevant component ***X***_rel_ and a task-irrelevant component ***X***_irr_. Although in this study, we relied on ridge regression to estimate ***w***, the following decomposition of the input data into task-relevant and task-irrelevant components can be applied in combination with any linear regression technique.

For performance prediction, the task-relevant component ***X***_rel_ should include all information inherent in the motion data ***X***. This requirement on the task-relevant component can be expressed as ${{\boldsymbol{X}}}_{{\rm{rel}}}{\boldsymbol{w}}={\boldsymbol{Xw}}$, which is satisfied when ***X***_rel_ minimizes the cost function

3 $$\frac{1}{2}{({\boldsymbol{Xw}}-{{\boldsymbol{X}}}_{{\rm{rel}}}{\boldsymbol{w}})}^{T}({\boldsymbol{Xw}}-{{\boldsymbol{X}}}_{{\rm{rel}}}{\boldsymbol{w}})$$

under the constraint ${\boldsymbol{X}}\ne {{\boldsymbol{X}}}_{{\rm{rel}}}$ to avoid the self-evident answer. The ***X***_rel_ that minimizes this cost function can be written as

4 $${{\boldsymbol{X}}}_{{\rm{rel}}}={\boldsymbol{Xw}}{{\boldsymbol{w}}}^{T}{({\boldsymbol{w}}{{\boldsymbol{w}}}^{T})}^{\dagger }={\boldsymbol{X}}\frac{{\boldsymbol{w}}{{\boldsymbol{w}}}^{T}}{|{\boldsymbol{w}}{|}^{2}},$$

where (**ww**^*T*^)^†^ is the pseudoinverse of **ww**^*T*^ and $|{\boldsymbol{w}}|=\sqrt{{{\boldsymbol{w}}}^{T}{\boldsymbol{w}}}$. The equality ${{\boldsymbol{w}}}^{T}{({\boldsymbol{w}}{{\boldsymbol{w}}}^{T})}^{\dagger }=\frac{{{\boldsymbol{w}}}^{T}}{|{\boldsymbol{w}}{|}^{2}}$ holds when ${\boldsymbol{w}}\in {{\rm{R}}}^{D\times 1}$. It is straightforward to confirm that ${{\boldsymbol{X}}}_{{\rm{rel}}}{\boldsymbol{w}}={\boldsymbol{Xw}}$, which indicates that ***X***_rel_ in equation (4) includes all the information relevant to performance prediction that is inherent in the motion data ***X***.

To investigate the properties of equation (4), especially the properties of the multiplication by $\frac{{\boldsymbol{w}}{{\boldsymbol{w}}}^{T}}{|{\boldsymbol{w}}{|}^{2}}$ on the right-hand side, we consider a vector and the decomposition of that vector as ${\boldsymbol{k}}={k}_{1}{{\boldsymbol{w}}}^{T}+{k}_{2}{{\boldsymbol{w}}}_{{\rm{ort}}}^{T}$, where ***w***_ort_ is a vector orthogonal to ***w*** (i.e., ${{\boldsymbol{w}}}_{{\rm{ort}}}^{T}{\boldsymbol{w}}=0$). The multiplication by $\frac{{\boldsymbol{w}}{{\boldsymbol{w}}}^{T}}{|{\boldsymbol{w}}{|}^{2}}$ on the right-hand side yields ${\boldsymbol{k}}\frac{{\boldsymbol{w}}{{\boldsymbol{w}}}^{T}}{|{\boldsymbol{w}}{|}^{2}}={k}_{1}{{\boldsymbol{w}}}^{T}$. Because ${k}_{1}{{\boldsymbol{w}}}^{T}$ is invariant and ${k}_{2}{{\boldsymbol{w}}}_{{\rm{ort}}}$ disappears after the multiplication, the multiplication by $\frac{{\boldsymbol{w}}{{\boldsymbol{w}}}^{T}}{|{\boldsymbol{w}}{|}^{2}}$ on the right-hand side represents the projection of the vector ***k*** onto ***w***. By applying this interpretation to each row of the matrix, this interpretation can be extended to the entire matrix. Equation (4) thus indicates that extracting the task-relevant components as part of the linear regression process is equivalent to projecting the data onto the weight vectors.

Under the decomposition ${\boldsymbol{X}}={{\boldsymbol{X}}}_{{\rm{rel}}}+{{\boldsymbol{X}}}_{{\rm{irr}}}$, ***X***_irr_ can be written as

5 $${{\boldsymbol{X}}}_{{\rm{irr}}}={\boldsymbol{X}}-{{\boldsymbol{X}}}_{{\rm{rel}}}={\boldsymbol{X}}({\boldsymbol{I}}-\frac{{\boldsymbol{w}}{{\boldsymbol{w}}}^{T}}{|{\boldsymbol{w}}{|}^{2}}),$$

where ${\boldsymbol{I}}\in {{\rm{R}}}^{D\times D}$ is the identity matrix. Under the appropriate normalization (i.e., with the mean and standard deviation of each component of ***X*** and ***d*** set to 0 and 1, respectively), ${{\boldsymbol{X}}}_{{\rm{rel}}}{\boldsymbol{w}}={\boldsymbol{Xw}}={\boldsymbol{h}}$ and ${{\boldsymbol{X}}}_{{\rm{irr}}}{\boldsymbol{w}}=0$, indicating that ***X***_rel_ and ***X***_irr_ denote the task-relevant and task-irrelevant components, respectively, under the framework of linear regression. An important feature of this framework is that it does not require any explicit function (e.g., forward kinematics, as in the UCM approach, or a task function, as in the GEM and TNC approaches); it requires only the data ***X*** and ***d***.

**Figure 1:**
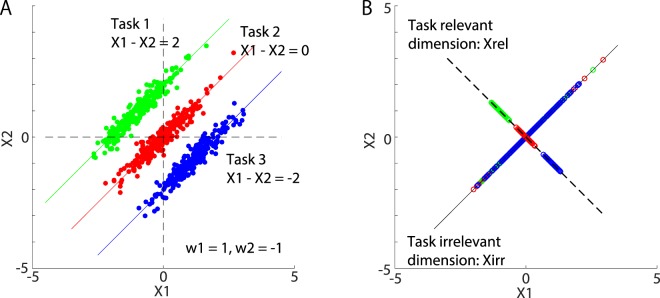
The concept of our method. (**A**) An example of decomposing input data ***X*** into the task-relevant component ***X***_rel_ and the task-irrelevant component ***X***_irr_. In this case, we assumed that in task 1, ${X}_{1}-{X}_{2}$ was required to be 2 (green line); in task 2, ${X}_{1}-{X}_{2}$ was required to be 0 (red line); and in task 3, ${X}_{1}-{X}_{2}$ was required to be −2 (blue line). Green, red, and blue dots represent the typical input data for tasks 1, 2, and 3, respectively. In the ridge regression process, these tasks can be achieved with ${w}_{1}=1$ and ${w}_{2}=-\,1$ (i.e., $h={w}_{1}{X}_{1}+{w}_{2}{X}_{2}={X}_{1}-{X}_{2}$ should be determined differently for each task). We randomly sampled the data shown as green dots from a two-dimensional Gaussian distribution whose mean and covariance matrix were $(\,-\,1,1)$ and $$(\begin{array}{cc}0.75 & 0.7\\ 0.7 & 0.75\end{array})$$, respectively. With the same covariance matrix, we randomly sampled the data shown as red and blue dots from two-dimensional Gaussian distributions whose means were (0, 0) and (1, −1), respectively. (**B**) The input data were decomposed into a task-relevant component ${{\boldsymbol{X}}}_{{\rm{rel}}}={\boldsymbol{Xw}}{{\boldsymbol{w}}}^{T}/|{\boldsymbol{w}}{|}^{2}$ (dotted black line) and a task-irrelevant component ${{\boldsymbol{X}}}_{{\rm{irr}}}={\boldsymbol{X}}-{{\boldsymbol{X}}}_{{\rm{rel}}}$ (solid black line). The data corresponding to ***X***_rel_ can be separated into different clusters depending on the task, whereas the ***X***_irr_ data show no such separation, thus demonstrating that this decomposition enables the investigation of both the task-relevant and task-irrelevant components.

Figures 1A and B present typical examples of the decomposition process when ***X*** includes only 2 elements and the task is constrained by setting $h={X}_{1}-{X}_{2}$ (i.e., ${w}_{1}=1$ and ${w}_{2}=-\,1$) to specific values (e.g., *y* = 2, 0, and −2 in the simulated tasks 1, 2, and 3, respectively). The simulated input data ***X*** were randomly sampled from a two-dimensional Gaussian distribution. Because the constrained task was one-dimensional and the input data were two-dimensional, an infinite pattern of ***X*** values resulted in identical *h* values. In this case, $${{\boldsymbol{X}}}_{{\rm{rel}}}={\boldsymbol{X}}\frac{1}{\sqrt{2}}(\begin{array}{cc}1 & -\,1\\ -\,1 & 1\end{array})$$ = $\frac{1}{\sqrt{2}}({X}_{1}-{X}_{2},-\,({X}_{1}-{X}_{2}))$ and ${{\boldsymbol{X}}}_{{\rm{irr}}}={\boldsymbol{X}}-{{\boldsymbol{X}}}_{{\rm{rel}}}$. The simulated data points on the dotted line in Fig. 1B correspond to ***X***_rel_; they can be clearly separated into three clusters corresponding to the simulated tasks 1, 2, and 3. In contrast, the data points plotted on the solid line, corresponding to ***X***_irr_, cannot be separated by task.

### Goal-directed whole-body movements and motor adaptation

**Figure 2:**
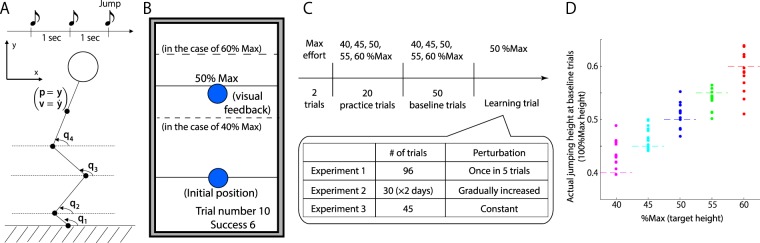
Summary of our experimental settings. (**A**) Three beeps were sounded at intervals of one second. Participants were instructed to perform a vertical jump at the time of the third beep. We measured and analyzed the joint angles at the toe, ankle, and knee in the sagittal plane. The jump height was measured based on the y-axis position of a marker attached to each participant’s back. (**B**) Task instructions and feedback information in each trial. A computer monitor was located in front of the participants (1.5 meters ahead, 1.7 meters above the floor). One second before the first beep, the target height (indicated by a black bar and text [e.g., 50% max]), baseline height (indicated by a black bar), and initial position (indicated by a blue cursor located at the baseline height) were visualized. When the target height was 60%, the black bar and text were displayed at the position of the higher black dotted bar. When the target height was 40%, the black bar and text were displayed at the position of the lower black dotted bar. These black dotted bars were provided solely during the task explanation; they were not visible during the experiments. In the practice trials, the blue cursor was visualized during the trials to continuously indicate the y-axis position of the marker attached to the subject’s back. These trials enabled the participants to become accustomed to the experimental setting. In the baseline and learning trials, the blue cursor was visualized at the beginning and end of each trial. At the beginning of each trial, the blue cursor was visualized at the baseline height. At the end of each trial, the cursor was visualized based on the actual jump height. When the jump height was close to the target height, the participants heard a coin-clattering sound. During the experiments, the subjects were provided with the current trial number and the number of successful trials (those in which they had heard the coin-clattering sound). (**C**) The sequence of experiments. The participants performed two trials of vertical jumps with maximum effort. The corresponding jump heights were used to determine the target heights. The participants underwent 20 practice trials, 50 baseline trials, and some number of learning trials, the number of which depended on the sspecific experiment. (**D**) Average jump height of each participant in the baseline trials of experiment 1. The jump height depended on the target height (one-way repeated measure ANOVA, p = 6.114 × 10^−24^), indicating that the participants were able to perform the desired goal-directed movement.

The current study focused on goal-directed and whole-body movements in which subjects manage to achieve the desired movements by controlling a large number of DoFs. We focused on a simplified type of whole-body movement: a vertical jump while crossing the arms in front of the trunk (Fig. 2A). This goal-directed whole-body movement enabled us to focus on lower limb and trunk motions to assess task-relevant variability, task-irrelevant variability, and the low-dimensional space in which a high proportion of the motor variability is embedded. We developed a machine learning technique to simultaneously evaluate these features of variability while considering how the motion changes over time and the relevance of each motion to the jump height.

Subjects stood in a fixed position and were instructed to look at a computer monitor located in front of them and perform a submaximum vertical jump with a given target height (40, 45, 50, 55, or 60% of the maximum jump height of the subject; see Fig. 2B). Three beeps were sounded, and the subjects were asked to perform the jump at the time of the third beep. The interval between beeps was one second. At the beginning of each trial (i.e., one second before the first beep), the target height was indicated by a black bar displayed on a computer monitor. At the end of the *t*th trial, the actual jump height *k*_*t*_ (the y position of the marker attached to the subject’s back) was displayed as a blue cursor on the monitor, where $t=1,\ldots ,T$ and *T* was the number of trials to be analyzed. By manipulating the displayed jump height (we refer to this manipulation as a perturbation *p*_*t*_), it was possible to induce sensory prediction error between the predicted and actual jump heights. This perturbation paradigm is similar to a protocol for motor gain adaptation that has been reported mainly for saccade and arm-reaching movements^31,32^. We expected the subjects to modify their motion to minimize the sensory prediction error.

First, we determined whether the subjects could perform goal-directed whole-body movements in our experimental setting. In the 50 baseline trials of experiment 1 (Fig. 2C), the target height was changed pseudorandomly in each trial. The target height was found to exert a significant main effect on the jump height (Fig. 2D, one-way repeated measure ANOVA, p = 6.114 × 10^−24^), indicating that the subjects could perform goal-directed vertical jumps dependent on the target height.

**Figure 3:**
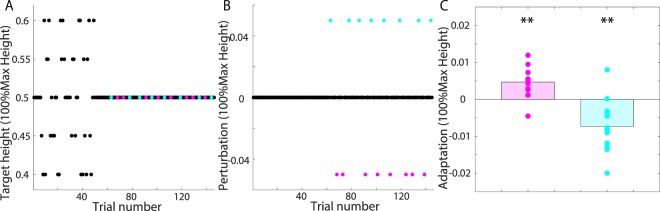
Diagram and results of experiment 1. (**A**) Target height in baseline and learning trials. The cyan and magenta circles indicate trials with perturbations. (**B**) Perturbation sequence. The cyan circles indicate the trials with ${p}_{t}=0.05$, and the magenta circles indicate those with ${p}_{t}=-\,0.05$. The perturbations were pseudorandomly imposed only once in every set of five trials. (**C**) Adaptation effect. The vertical line indicates the difference between the jump height in the next trial after a perturbation and that in the trial in which the perturbation was imposed. The magenta dots indicate the average difference for each subject corresponding to the perturbation ${p}_{t}=-\,0.05$, and the cyan dots indicate the average difference for each subject corresponding to the perturbation ${p}_{t}=0.05$.

Second, we determined whether the subjects showed motor adaptation in our experimental setting. In the 96 learning trials of experiment 1 (Fig. 2C), the subjects experienced a perturbation once in every set of five trials; the perturbation was pseudorandomly set to ${p}_{t}=0.05$ or ${p}_{t}=-\,0.05$ in one trial of every five and was set to ${p}_{t}=0$ in the other trials (Fig. 3A and B). We observed a modification in jump height after each perturbation (Fig. 3C, paired t-test, $p=0.0026$ for motor adaptation when ${p}_{t}=0.05$ and $p=0.0014$ for motor adaptation when ${p}_{t}=-\,0.05$). No statistically significant modification of the jump height was observed either two trials after the perturbation (paired t-test, $p=0.0528$ for motor adaptation when ${p}_{t}=0.05$ and $p=0.2875$ for motor adaptation when ${p}_{t}=-\,0.05$) or three trials after the perturbation (paired t-test, $p=0.7407$ for motor adaptation when ${p}_{t}=0.05$ and $p=0.4528$ for motor adaptation when ${p}_{t}=-\,0.05$). Additionally, we investigated whether fatigue influenced the adaptation by comparing the magnitudes of the modifications in the earlier learning trials to those in the later trials and found no significant difference (paired t-test, $p=0.4382$). Motor adaptation could thus be observed in the goal-directed vertical jumping task without a significant fatigue effect.

### Validation of ridge regression and decomposition into task-relevant and task-irrelevant components

The current study focused on the evaluation of motor variability (especially task-relevant variability, task-irrelevant variability, and the relevance of a low-dimensional structure to task performance) through an extension of ridge regression. Before performing the variability evaluation, we needed to validate the ridge regression procedure in the current experimental setting. Notably, we have already validated the efficiency of ridge regression for predicting performance not only in jumping movements but also in throwing movements^30^.

**Figure 4:**
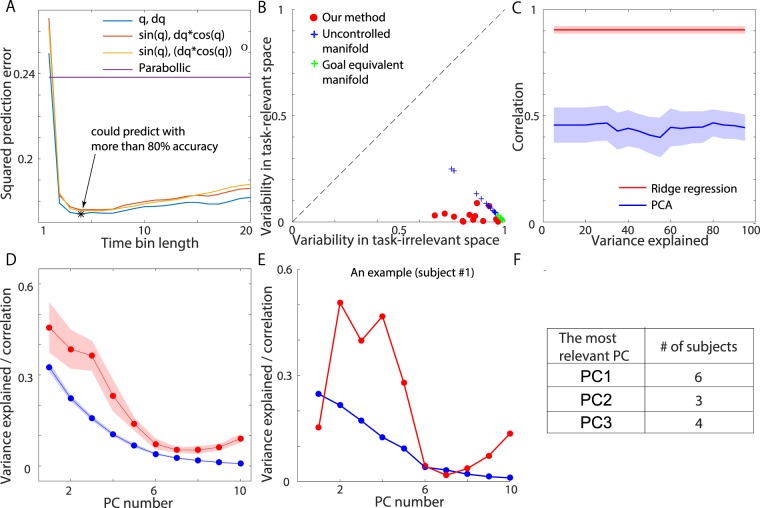
Validation of our method and comparison to previous methods. (**A**) Predictive power of ridge regression using three kinds of input data. The time bin length used for the ridge regression procedure and the squared prediction error are presented on the horizontal and vertical axes, respectively. A prediction error of 1 corresponds to a case in which ridge regression is unable to yield a prediction, and a prediction error of 0 corresponds to a case in which ridge regression is able to predict the output data perfectly. The results indicate that ridge regression was capable of predicting the output data with an accuracy of 82.6 ± 2.28% (mean ± SEM, N = 13). (**B**) Evaluation of task-relevant and task-irrelevant variabilities. The red dots indicate the variabilities evaluated using our method for each subject (N = 13). The blue and green crosses indicate the variabilities evaluated using the UCM and GEM methods, respectively. Our method uses a normalization method different from that of the UCM and GEM approaches. (**C**) Correlation between the predicted and actual jump heights. The red line and shaded area represent the mean and SEM, respectively, of the correlation for ridge regression (N = 13). The blue line and shaded area represent the mean and SEM, respectively, of the correlation for PCA (N = 13). The explained variance or the corresponding number of PCs and the correlation are presented on the horizontal and vertical axes, respectively. (**D**) The explained variance and the correlation between the predicted and actual jump heights for each PC. The red line and shaded area represent the mean and SEM, respectively, of the correlation (N = 13). The blue line and shaded area represent the mean and SEM, respectively, of the amount of variance explained (N = 13). (**E**) The explained variance and the correlation between the predicted and actual jump heights for each PC for a typical subject. The red and blue lines represent the correlation and the explained variance, respectively. (**F**) The number of subjects for which each of the first three PCs was the most relevant for predicting the jump height.

Ridge regression requires careful selection of the input data, which is indispensable for assessing the linear relation between the input and output data. The prediction power is a sophisticated measure for selecting input data while avoiding overfitting^28^. The current study focused on the prediction error between the actual and predicted jump heights using 10-fold cross-validation. We compared three types of candidate input data. The first candidate data type consisted of joint angles {*q*_*i*_} and angular velocities $$\{{\dot{q}}_{i}\}$$: $${\boldsymbol{X}}=(\{{q}_{i}\},\{{\dot{q}}_{i}\})$$, where $$\{{q}_{i}\}=({q}_{1},{q}_{2},{q}_{3},{q}_{4})$$ and $${\dot{q}}_{i}$$ denotes the derivative of *q*_*i*_ with respect to time (the definition of each *q*_*i*_ is given in Fig. 2A). The second candidate data type consisted of the functions *q*_*i*_ and $${\dot{q}}_{i}$$, which describe the position and velocity, respectively, of the marker on the subject’s back on the y-axis and are relevant to the jump height: $${\boldsymbol{X}}=(\{\,\sin \,{q}_{i}\},\{{\dot{q}}_{i}\,\cos \,{q}_{i}\})$$. The third candidate data types consisted of functions describing the jump height based on a parabolic approximation: $${\boldsymbol{X}}=(\{\,\sin \,{q}_{i}\},\{{\dot{q}}_{i}{\dot{q}}_{j}\,\cos \,{q}_{i}\,\cos \,{q}_{j}\})$$, where $$\{{a}_{i}{a}_{j}\}=({a}_{1}^{2},{a}_{1}{a}_{2},{a}_{1}{a}_{3},{a}_{1}{a}_{4},{a}_{2}^{2}$$, $$,{a}_{2}{a}_{3},{a}_{2}{a}_{4},{a}_{3}^{2},{a}_{3}{a}_{4},{a}_{4}^{2})$$. By comparing these three candidates, we found that the first candidate (i.e., $${\boldsymbol{X}}=(\{{q}_{i}\},\{{\dot{q}}_{i}\})$$) resulted in the lowest prediction error (Fig. 4A). More specifically, the first candidate yielded the lowest prediction error when data corresponding to the last four time frames before release were considered. A prediction error of 1 indicates that the method cannot predict the output data at all. In contrast, a prediction error of 0 indicates that the method can predict the output data with 100% accuracy. As shown in Fig. 4A, the first candidate data type with four time frames of data resulted in a prediction error of 0.174, indicating that ridge regression enabled prediction of the jump height with an accuracy of 82.6 ± 2.28% (mean ± standard error of the mean (SEM), N = 13) in the current setting. Thus, in the following, we refer to the first candidate data type with four time frames of data as the motion data. For our experimental setting with a goal-directed vertical jump task, ***X*** included 32 dimensions for each trial (4 dim × 4 time frames for {*q*_*i*_} and 4 dim × 4 time frames for $$\{{\dot{q}}_{i}\}$$). Because the parabolic approximation of the jump height based on the y-axis position *p* and velocity *v* of the marker on the subject’s back at the release time (i.e., $$h=p+\tfrac{{v}^{2}}{2g}$$) enabled prediction of the jump height with an accuracy of 76 ± 2.96% (mean ± SEM., N = 13), as seen from the purple line in Fig. 4A, it is clear that the ridge regression approach enables jump height prediction with higher accuracy compared with this approximation. The ridge regression approach shows higher prediction power due to its robustness against observation noise and its consideration of how the motion changes over time rather than only representative motion data from a single time frame (i.e., the position and velocity of the hip joint only at the time of release). Ridge regression thus enables the investigation of the linear relation between the time-varying motion and the jump height with appropriate precision.

### Variability in task-relevant and task-irrelevant space

We calculated the task-relevant and task-irrelevant variabilities in goal-directed vertical jumps based on both ridge regression and the decomposition of the input data into task-relevant and task-irrelevant dimensions. In the current study, the variability (variance) of each element of ***X***_rel_ and ***X***_irr_ in focused trials was calculated. Representative values of the variability, the task-relevant variability Var_rel_ and the task-irrelevant variability Var_irr_ were calculated by averaging the variability across all dimensions.

We found that the task-relevant variability was smaller than the task-irrelevant variability for all participants (N = 13, red dots in Fig. 4B). The results of previous methods, such as the UCM method (blue crosses) and the GEM method (green crosses), indicated similar task-relevant and task-irrelevant variabilities; however, our method enabled the extraction of a lower task-relevant variability and a higher task-irrelevant variability. Because the normalization procedures of our method and previous methods differ, slight differences were observed in the calculated variabilities. Our method enables both the task-relevant and task-irrelevant variabilities to be quantified by considering how the motion changes over time and its relevance to the task of interest. Our method does not require any explicit task function, such as the parabolic approximation of jump height; instead, it determines the relevance of the motion to the task in a data-driven manner. Furthermore, our method is robust against observation noise due to the properties of ridge regression.

### Relevance of each principal component to task performance

Movement variability exhibits not only less task-relevant variability than task-irrelevant variability but also a low-dimensional structure (i.e., a large proportion of the movement variability is embedded in a limited number of dimensions). In the current study, we compared our method against PCA, which is a conventional method for extracting low-dimensional structures. Because the low-dimensional structure is considered to represent certain features of motor control, it can be expected to be correlated with task performance. We decomposed the motion data ***X*** into principal components (PCs, i.e., eigenvectors) and calculated the correlation between each PC and the jump height. The PCs compose a set of orthonormal bases inherently contained in ***X***. The PCs that are identified earlier in the decomposition process (e.g., the 1st PC) explain a larger proportion of the variance inherent in ***X*** than do the PCs that are identified later (e.g., the 1st PC explains a larger proportion of the variance inherent in ***X*** than does the 2nd PC, the 2nd PC explains a larger proportion of the variance than does the 3rd PC, etc.). In our setting, no clear relation was found between the number of PCs identified during the decomposition process and the correlation between the decomposed motion data and performance data (Fig. 4C). When averaged across all participants, the 1st PC explained approximately 30% of the movement variability (blue line in Fig. 4D). Corresponding to the explained movement variability, the 1st PC also showed the highest correlation with jump height (red line in Fig. 4D) when averaged across all participants. For the particular subject represented in Fig. 4E, however, the 2nd rather than the 1st PC showed the highest correlation with the jump height (see the red line in this figure). This subject was not a special case; Fig. 4F shows the numbers of subjects for which each of the first three PCs showed the highest correlation with the jump height. For 6 out of 13 subjects, the 1st PC showed the highest correlation with performance; however, for 3 subjects, the 2nd PC showed the highest correlation, and for 4 subjects, the 3rd PC showed the highest correlation. These results demonstrate that the amount of movement variability explained by a PC did not directly correspond to its relevance to task performance for individual subjects.

Ridge regression (the red line in Fig. 4C) enabled jump height prediction with higher accuracy compared with PCA because in ridge regression, each PC is weighted based on both the explained movement variability and the task relevance, as follows. In PCA (or, equivalently, in singular value decomposition (SVD)), the time-varying motion in the *t*th trial is decomposed using a set of orthonormal basis functions $({{\boldsymbol{v}}}_{1},{{\boldsymbol{v}}}_{2},\ldots ,{{\boldsymbol{v}}}_{N})$ as follows: ${{\boldsymbol{X}}}_{t}={\sum }_{i=1}^{N}\,{\lambda }_{i}{u}_{i,t}{v}_{i}$, where *N* is the number of PCs, *λ*_*i*_ is the *i*th eigenvalue corresponding to the *i*th PC (***v***_*i*_), *u*_*i*,*t*_ indicates the proportion of the motion data from the trial explained by the *i*th PC, and *λ*_*i*_ is proportional to the variance explained by the *i*th PC. Because PCs identified earlier explain a larger proportion of the variance than do PCs identified later, ${\lambda }_{1}\ge {\lambda }_{2}\ge \cdots \ge {\lambda }_{N}\ge 0$. The correlation between the trial-to-trial variance contribution of the *i*th PC and the trial-to-trial variance in task performance was thus calculated based on *u*_*i*,*t*_. Notably, *u*_*i*,*t*_ reflects the contribution of the *i*th PC in explaining motion data rather than the relevance of the *i*th PC to task performance. Thus, the 1st PC is not necessarily the PC that is most relevant to performance in a framework based on PCA or SVD. In contrast, ridge regression enables the prediction of task performance as follows: ${h}_{t}={\sum }_{i=1}^{N}\,f({\lambda }_{i})\,{\rm{Corr}}({u}_{i,t},{d}_{t}){u}_{i,t}$, where *f*(*λ*_*i*_) is a sigmoidal function of *λ*_*i*_ and ${\rm{Corr}}({u}_{i,t},{d}_{t})$ is the correlation between the contribution of the *i*th PC in the *t*th trial (*u*_*i*,*t*_) and the observed jump height (*d*_*t*_); see the *Materials and Methods* section for the detailed mathematical calculations. Ridge regression thus enables the prediction of performance by weighting each PC based on both the explained movement variability and the task relevance through *f*(*λ*_*i*_) and ${\rm{Corr}}({u}_{i,t},{d}_{t})$, respectively. In other words, our method enables the consideration of the low-dimensional structure of movement variability by weighting each PC in a manner suitable for predicting task performance.

### Influence of motor adaptation on variability in the task-relevant and task-irrelevant dimensions

One advantage of our method is its linearity, which enables the simultaneous comparison of the task-relevant and task-irrelevant variabilities under conditions in which the mean kinematics or task parameters are changing (e.g., before, during, and after motor learning). It was previously unclear how task-relevant and task-irrelevant variabilities are modulated by motor adaptation. The modulation of these variabilities has been investigated for arm-reaching movements and for motor adaptation in response to a constant perturbation^5,33^. Although some differences exist between adaptation to a constant perturbation and adaptation to a gradually imposed perturbation (e.g., retention rate or awareness^34^), the means by which those variabilities are modulated in the two types of adaptation have not been investigated. Furthermore, it was previously unclear whether such modulation of variability could be observed in whole-body movements. Our method, with its lack of linear approximations, can enable the investigation of how the task-relevant and task-irrelevant variabilities are modulated before and after motor adaptation in whole-body movements. We thus applied our method to investigate motor adaptation in response to both constant and gradually imposed perturbations.

**Figure 5:**
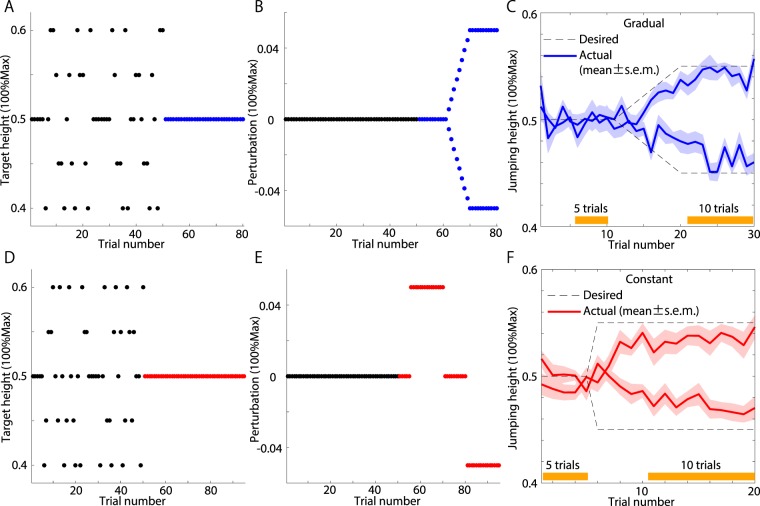
Diagrams and results of experiments 2 and 3. (**A**,**D**) Target height in baseline and learning trials. The blue and red circles indicate trials with perturbations. (**B**,**E**) Perturbation sequence. The subjects participated in experiment 2 over two days and experienced two different perturbations (either $p > 0$ or $p < 0$). The order of the perturbations was counterbalanced across subjects. In experiment 3, the subjects experienced both positive and negative perturbations within the same day. Although panel (E) shows only the case in which a negative perturbation followed a positive one, the order of the perturbations was counterbalanced across subjects. (**C**,**F**) Learning curves. The thin solid lines represent the learning curves of each subject. The bold solid lines represent the learning curves averaged across all subjects. Orange bars indicate the trials before and after adaptation for calculating task-relevant and task-irrelevant variabilities.

In experiment 2 (two days for each subject), the subjects experienced gradually increasing or decreasing perturbations. Each subject underwent ten learning trials without any perturbation. Then, a perturbation of 0.05 or −0.05 was gradually imposed over ten trials (Fig. 5A and B). This gradually imposed perturbation required gradual rather than abrupt compensation (i.e., the subjects were required to modify their motions slightly in each trial). In a total of 30 trials, the target height was set to 50% of the subject’s maximum jump height. Subjects who experienced a ${p}_{t} > 0$ on the first day experienced a ${p}_{t} < 0$ on the 2nd day and vice versa. The order of the perturbations was counterbalanced across subjects. It was found that the subjects were able to adapt to the gradually increasing or decreasing perturbations (Fig. 5C).

In experiment 3, the subjects experienced constant perturbations. Each subject underwent five learning trials without any perturbation. Then, the perturbation was set to 0.05 or −0.05 for 15 trials, to 0 for the subsequent ten trials for washout and to −0.05 or 0.05 for the final 15 trials (Fig. 5D and E). In contrast to experiment 2, in which the perturbation was gradually imposed, the subjects were required to abruptly modify their motions in experiment 3. Subjects who experienced a ${p}_{t}=0.05$ in the 6th–20th trials experienced a ${p}_{t}=-\,0.05$ in the 31st–45th trials and vice versa. The order of the perturbations was counterbalanced across subjects. In a total of 45 trials, the target height was set to 50% of the subject’s maximum jump height. In both experiments 2 and 3, the subjects adapted to the perturbations (Fig. 5F).

**Figure 6:**
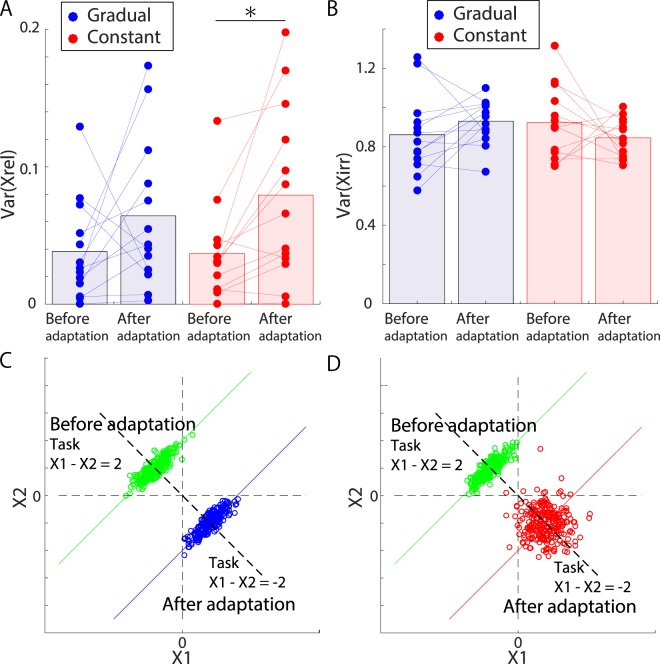
Application of our method to the results of experiments 2 and 3. (**A**) Task-relevant variabilities of each subject (N = 13) before and after adaptation to perturbation in experiments 2 and 3. The blue and red dots represent the variabilities of each subject in experiments 2 and 3, respectively. The blue and red lines show the modulation of the variability due to adaptation. The blue and red bars indicate the variabilities averaged across all subjects. There was a significant difference between the preadaptation and postadaptation variabilities in experiment 3 (Wilcoxon signed rank test, p = 0.0034). (**B**) Task-irrelevant variabilities of each subject (N = 13) before and after adaptation in experiments 2 and 3. (**C**,**D**) Interpretation of our results based on a simple example. We assume that in the task before adaptation, ${X}_{1}-{X}_{2}$ was required to be 2, and in the task after adaptation, ${X}_{1}-{X}_{2}$ was required to be −2. Panel (C) presents an interpretation of our results for experiment 2. In experiment 2, there was no modulation in either the task-relevant or task-irrelevant variabilities. Panel (D) suggests an explanation of our findings from experiment 3. In that experiment, the task-relevant variabilities increased after adaptation, whereas the task-irrelevant variabilities remained unchanged.

We calculated the task-relevant and task-irrelevant variabilities before and after adaptation in experiments 2 and 3 (Fig. 6A and B). For the task-relevant variability, there was no significant difference before and after adaptation to gradually increasing or decreasing perturbations (blue dots in Fig. 6A, N = 13, Wilcoxon signed rank test, p = 0.1909). In contrast, when adapting to a constant perturbation, there was a significant difference in the task-relevant variability before and after adaptation (red dots in Fig. 6A, N = 13, Wilcoxon signed rank test, p = 0.0034). For the task-irrelevant variability, no significant difference before and after adaptation was observed for either a gradually increasing or decreasing perturbation (blue dots in Fig. 6B, N = 13, Wilcoxon signed rank test, p = 0.1677) or a constant perturbation (red dots in Fig. 6B, N = 13, Wilcoxon signed rank test, p = 0.3396). These results can be interpreted based on a simulated two-dimensional case similar to that shown in Fig. 1 (Fig. 6C and D). When adapting to perturbations, the subjects needed to modify their outputs (i.e., jump heights) by determining appropriate inputs (i.e., motion data). When adapting to gradually increasing or decreasing perturbations, there was no modulation in either the task-relevant or the task-irrelevant variability (Fig. 6C). In contrast, when adapting to a constant perturbation, the task-relevant variability increased, but the task-irrelevant variability was not modulated (Fig. 6D). Notably, Fig. 6C and D do not show real data; they are simulated examples used to interpret our results. In summary, the modulation of the task-relevant variability depends on the perturbation schedule.

## Discussion

We have proposed a flexible and straightforward machine learning technique for decomposing motion that changes over time into task-relevant and task-irrelevant components, quantifying task-relevant and task-irrelevant variabilities, and determining the relevance of each PC to task performance in a noise-robust manner while considering how motion changes over time and its relevance to task performance (Fig. 4). Our method can identify the relevance of time-varying motion to performance (i.e., the task function) in a data-driven manner without requiring any explicit task function, such as the parabolic approximation of jump height. Furthermore, our method does not require any linear approximation, and thus, it enables simultaneous consideration of the variabilities when the kinematics or task parameters are changing across trials (e.g., before, during, and after adaptation). By applying our method to motion data collected before and after motor adaptation, we found that the perturbation schedule affects the modulation of movement variability in motor adaptation (Fig. 6A and B). These advantages enable our method to be flexibly applied to a wide range of goal-directed movements.

Our method can be regarded as a generalized version of the UCM and GEM methods. When we define ${X}_{i}={q}_{i}-{\bar{q}}_{i}$ (where *q*_*i*_ and ${\bar{q}}_{i}$ denote the *i*th joint angle and the joint angle averaged across all trials, respectively [$i=1,\ldots ,4$ in our setting]) and ${X}_{i+4}={\dot{q}}_{i}-{\bar{\dot{q}}}_{i}$ (where ${\dot{q}}_{i}$ and ${\bar{\dot{q}}}_{i}$ denote the angular velocity of the *i*th joint and the joint angular velocity averaged across all trials, respectively) and the corresponding weight matrix ***w*** is the Jacobian matrix of forward kinematics with $p=p({\boldsymbol{q}})$ (the back position) and $v=v({\boldsymbol{q}},\dot{{\boldsymbol{q}}})$ (the back velocity) around the average joint angles and angular velocities across all trials ($\bar{{\boldsymbol{q}}}$ and $\bar{\dot{{\boldsymbol{q}}}}$), our framework corresponds to the UCM framework. When we define ${w}_{1}=1$, ${w}_{2}=\frac{\bar{v}}{g}$, ${X}_{1}=p-\bar{p}$, ${X}_{2}=v-\bar{v}$, and $h={\boldsymbol{Xw}}=p-\bar{p}+\frac{\bar{v}}{g}(v-\bar{v})$, our framework corresponds to the GEM framework for cases in which the task function can be defined as a parabolic function, where *g* denotes the gravitational acceleration. Because the UCM and GEM methods can be regarded as special cases of our method, our method can be regarded as a generalized version of those methods.

Another advantage of our method is the ability to select appropriate input data based on the corresponding predictive power (Fig. 4A). Furthermore, there is a possibility that the predictive power could also enable the selection of proper coordinates in which to define the task performance. A previous study has demonstrated that the UCM and TNC frameworks are sensitive and insensitive, respectively, to how the coordinate system is selected (e.g., using either a relative or absolute angle)^35^. Our framework is likely sensitive to how the coordinates are chosen; however, in contrast to the UCM framework, our method enables the selection of appropriate coordinates for investigating the relationship between motion and performance data based on predictive power. Although we considered one-dimensional (i.e., jump height) performance in the current study, investigating two-dimensional performance requires the definition of an appropriate coordinate system^30,36^. The predictive power plays a vital role in selecting the proper coordinates not only in the motion space but also in the performance space^30^. Selecting the length of the time frames is another crucial problem (Fig. 4A). In this study, the motion data from four time frames (approximately 33 ms) were selected because that length resulted in the best predictive power. Notably, our method can be applied independently of the length of the time frames. In our case, four time frames were chosen to maximize the predictive power. Those four-frame motion data represent dependent variables; however, ridge regression can be used to analyze dependent variables to interpret their dependence on certain target variables^29^. For prediction, the ridge regression approach relies on the appropriate weighting of PC vectors, which are orthogonal to each other, thus resolving the multicollinearity introduced by considering dependent variables. Notably, choosing a proper coordinate system is an important but difficult problem. Thus, our method is useful within the framework of regression methods.

For a regression analysis, it is necessary to pool data that can be regarded as representing the same condition (i.e., stationarity is essential). When analyzing the data from experiments 2 and 3, we analyzed motion data collected before, during, and after adaptation, which would seem, at a glance, to exhibit nonstationarity. To address this nonstationarity, we estimated appropriate linear coefficients ***w*** based on both training and learning trials. In the training trials, the subjects performed vertical jumps aiming for either 40%, 45%, 50%, 55%, or 60% of their maximum jump heights. In the learning trials, the subjects performed vertical jumps aiming for 50% of their maximum heights before adaptation, for heights from 50% to 45% of the maximum during adaptation to a positive applied perturbation, and for 45% of their maximum heights after adaptation (the same applies for a negative applied perturbation except that the value of 45% should be replaced with 55%). Thus, the motions to be performed in the training trials included the sequences before and after adaptation (aiming for either 45% or 55% of the maximum jump height). During adaptation, the subjects needed to perform jumps toward intermediate targets, such as 51% or 48% of the maximum height. Although motion data corresponding to those intermediate targets were not included in the training trials, the regression framework worked well for interpolation. Because the weight values ***w*** were estimated based on motion data from jumps aiming for 40%, 45%, 50%, 55%, and 60% of the maximum height, it was possible to use interpolation to predict the motions for 51% or 48% of the maximum jump height. In summary, when applying a regression framework to investigate adaptation, it is necessary to include motion data for the targets both before and after motor adaptation when estimating the weight values.

Although we compared our method to the UCM and GEM methods (Fig. 4B), we should also compare it to the TNC method^13,37^, the other main method used to quantify motor variability from a different perspective. The TNC method enables the extraction of three types of information from motion data: the T-cost, which quantifies how the mean motion data deviate from the optimal motion; the N-cost, which quantifies how the motor variability deviates from the optimal variability; and the C-cost, which quantifies how the covariance among the motion data deviates from the optimal covariance. Although the TNC method does not consider task-relevant and task-irrelevant variabilities, it can be used to quantify other interesting features (i.e., the T-, N-, C-costs) embedded in motion data and the variations of those costs during the learning process. Due to its computational cost, we could not apply the conventional version of the TCN method to our data. The TCN method requires a grid search for the calculation of the T-cost. Because the number of grids was 200 and the number of variables of interest was eight in our case (four joint angles and angular velocities), this method would require 200^8^ calculations. Due to this overly burdensome computational cost for calculating not only the T-cost but also the C-cost, we were unable to apply the TNC method to our case. However, when the number of variables of interest is two, the TNC method can be promising and can work well^13,37^. Notably, a potential future topic of research might be to apply optimization techniques to calculate the T- and C-costs. This approach could overcome the computational burden of the conventional TNC framework, thus making it suitable for various cases.

One potential extension of our method is to obtain a state-space model for motor adaptation during whole-body movements. A state-space model of motor adaptation was previously proposed, mainly for arm-reaching movements^38–46^. In the current study, the modification of the jump height in the *t*th trial, ${h}_{t}-{h}_{t-1}$, was significantly correlated with the error, *e*_*t*−1_, caused by perturbation and motor noise (in experiment 1, the correlation between ${h}_{t}-{h}_{t-1}$ and *e*_*t*−1_ averaged across all participants was 0.5118, with $p < 0.01$ for all participants). A state-space model of the jump height can thus be written as ${h}_{t}={h}_{t-1}+\eta {e}_{t-1}$, where $\eta$ (>0) is the learning rate. The jump height *h*_*t*_ is predicted well by ${h}_{t}\simeq {{\boldsymbol{X}}}_{t}{\boldsymbol{w}}={{\boldsymbol{X}}}_{{\rm{rel}},{\rm{t}}}{\boldsymbol{w}}$, enabling us to approximate the state-space model as ${{\boldsymbol{X}}}_{{\rm{rel}},{\rm{t}}}{\boldsymbol{w}}={{\boldsymbol{X}}}_{{\rm{rel}},{\rm{t}}-{\rm{1}}}{\boldsymbol{w}}+\eta {e}_{t-1}$. This model indicates that the jump height is modified by modifying the motion in the dimension along ***w***. Because this is a possible future extension of our approach, we will need to further investigate the frameworks mentioned above in addition to performing state-space modeling of the modulation of task-relevant and task-irrelevant variabilities, as mentioned in a previous study^38^.

An advantage of our method is its linearity (i.e., ${\boldsymbol{h}}={\boldsymbol{Xw}}$), in contrast to the nonlinearity inherent in body dynamics. A likely explanation for why linear regression works well can be found through analogy with the motor primitive framework, which is a framework that has been successfully used for motor adaptation in goal-directed arm-reaching movements^39–47^. In this framework, a nonlinear motor command *u* is modeled as a linear weighted sum of nonlinear neural activities ***A***, where $u={\sum }_{i}\,{W}_{i}{A}_{i}$ and the *W*_*i*_ are modified to minimize the movement error between the actual hand position and the desired movement position. When the *A*_*i*_ are nonlinear functions of the desired movement and are appropriately high-dimensional, nonlinear motor commands can be generated by means of appropriate linear combinations of these nonlinear neural activities, as theoretically demonstrated in the framework of a basis function network^48^. The motion data ***X*** can be nonlinearly related to movement performance because the human body exhibits nonlinear dynamics. Additionally, the motion data are appropriately high-dimensional (32 dimensions for a 1-dimensional task). Thus, an appropriate linear sum ***Xw*** can be used to predict the actual movement performance, resulting in an appropriately estimated ***w*** that represents the relevance of the motion elements to the performance.

In this study, we relied on simple linear regression (i.e., ridge regression); however, it is possible to use a more complicated machine learning technique, such as a mixture model^28,49–51^, a sparse regression technique^52^, or a nonlinear regression technique^53^. We have shown that a nonlinear regression technique such as Gaussian process regression is not effective for predicting performance based on motion data^30^, likely because of the limited number of data points. Although sparse regression, nonlinear regression, or a mixture model can generally achieve better predictive performance when the number of data points is sufficiently high, it is difficult to find the specific relations between the PCs and the estimated parameters via such methods. Ridge regression enables the determination of not only the task-relevant and task-irrelevant variabilities but also the relevance of each PC to the performance. Because some previous studies have also discussed the relevance of each PC to performance^54,55^, a promising research topic might be to evaluate the functional roles of such low-dimensional structures from a different viewpoint.

To our knowledge, only a few studies have investigated how variability is modulated throughout motor adaptation^5,33^. A previous study confirmed that the variability was modulated after motor adaptation in response to a constant force field^33^; however, to our knowledge, whether the perturbation schedule affected the modulation was not clarified. The current study suggests that the perturbation schedule does affect the modulation of variability (Fig. 6A and B). Because variability can facilitate exploration^33^ (cf. not only exploration but other various factors^56^), the current study also suggests that constant perturbations motivate exploration in the task-relevant space, whereas gradually applied perturbations do not affect exploration. Within the framework of reinforcement learning, exploration is expected to increase after the detection of novel phenomena or environmental changes in order to maximize the expected future reward (i.e., exploration bonus)^57^. Under a gradually applied perturbation, however, it is difficult to notice the existence of the perturbation^34,58^; thus, no need for a change in exploration behavior is perceived. In contrast, under an abruptly applied perturbation, it is easier to notice the existence of the perturbation^34,58^, resulting in a change in exploration behavior to maximize the future reward. Additionally, recent studies have suggested that variability plays an essential role in sports performance^59^, injury prevention^60^, and the development of children with developmental coordination disorders^61^. The current findings provide some hints regarding how these functions might be assisted through the encouragement of exploration via false feedback.

## Materials and Methods

### Participants

Thirteen healthy volunteers (aged 18–22 years, two females) participated in all of our experiments, which were approved by the ethics committee of the Tokyo University of Agriculture and Technology and were performed in accordance with guidelines and regulations. All the participants were informed of the experimental procedures in accordance with the Declaration of Helsinki, and all participants provided written informed consent before the start of the experiments.

On the first day, the participants underwent ten practice trials and 160 baseline trials with pseudorandomly changing targets (40%, 45%, 50%, 55%, or 60% of the maximum jump height) to familiarize them with the experimental setting. On the second, third, fourth, and fifth days (not consecutive), they participated in experiments 1, 2, and 3. Experiment 2 lasted for two days.

### Data acquisition and processing

The jumping motions were recorded at 120 Hz using six cameras (Optitrack Flex 13, NaturalPoint Inc., Corvallis, Oregon). Markers were attached to each participant’s back (TV10), right hip joint (femur greater trochanter), right knee (femur lateral epicondyle and femur medial epicondyle), right heel (fibula apex of the lateral malleolus and tibia apex of the medial malleolus), and right toe (head of the 2nd metatarsus). The marker position data were filtered with a 12th-order, 10 Hz zero-phase Butterworth filter using MATLAB 2016a. The joint angles between the right toe and heel (*q*_1_), right heel and shank (*q*_2_), right shank and thigh (*q*_3_), and right thigh and trunk (*q*_4_) were calculated in the sagittal plane (Fig. 2A). Because the current study focused on a vertical jump with the arms crossed in front of the trunk, it was possible to focus only on lower limb and trunk motions. Throughout the current study, we focused on a four-link model of the lower limbs in the sagittal plane.

The time of release was detected based on the moment at which the vertical toe position exceeded 10% of the maximum height in each trial. The predictive power was calculated using various time-bin lengths including the time of release (Fig. 4A). When the time bin length was four, the fourth time frame corresponded to the time of release, the third time frame corresponded to one time frame before the time of release, and the other time frames were ordered accordingly.

### Experimental setup

At the beginning of each trial, the subjects were instructed to stand in a fixed position. In each trial, the subjects listened to three beeps separated by one-second intervals; the first beep indicated the start of the trial, and the subjects were required to jump at the time of the third beep.

We measured the position of the marker attached to each subjects back using MATLAB at 30 Hz, which was the highest sampling frequency available using the MATLAB Optitrack software plugin, while displaying the measured position on a monitor with the same refresh rate as the measuring frequency. A monitor in front of the subject (1.5 meters ahead, 1.7 meters above the floor) displayed a blue cursor that indicated the height of the marker attached to the subjects back and a black bar that indicated the target height (Fig. 2B). The cursor and bar were displayed one second before the first beep sounded. The blue marker moved only along the vertical axis because the current study focused on the vertical height of the jumping motion. The marker position at time *s* on the y-axis (Fig. 2A), *k*_*s*_, was displayed on the monitor after being normalized for each subject as follows: ${k}_{s}=\frac{{\hat{k}}_{s}-{k}_{0}}{{k}_{{\rm{\max }}}-{k}_{0}}$, where ${\hat{k}}_{s}$ is the marker position without normalization, *k*_0_ is the initial marker position as evaluated in an upright standing position in each trial, and *k*_max_ is the jump height corresponding to the maximum effort of the subject (Fig. 2C). No cursor feedback was supplied for the two trials in which the subjects were required to jump with the maximum effort. One second before the first beep, a blue circle at ${k}_{s}=0$ was displayed on the black baseline on the monitor. Additionally, the target height *d* was indicated by a black line. Before the baseline trials, the subjects underwent ten practice trials. In those trials, the marker position in each time frame was displayed on the monitor, and *d* was pseudorandomly chosen from among 0.40, 0.45, 0.50, 0.55, and 0.60 (each value was randomly chosen only once in every set of five trials). This method enabled each subject to become acquainted with the experimental setting by confirming the motion trajectory of the marker attached to his or her back. In the baseline trials, the marker position was displayed only at the start and end of each trial. One second before the first beep, the cursor was displayed at the baseline position, and a black line corresponding to *d* was displayed based on a pseudorandom choice of 0.40, 0.45, 0.50, 0.55, or 0.60. At the end of each trial, the cursor was displayed at the maximum value of *k*_*s*_ reached in that trial (i.e., max *k*_*s*_), indicating the jump height (Fig. 2B). When the subjects achieved a jumping motion that was close to the target height ($|d-\,{\rm{\max }}\,{k}_{s}| < 0.02$), they heard a coin-clattering sound to indicate that the jumping motion was successful. After the baseline trials, the subjects underwent 96 learning trials in experiment 1, 30 trials in experiment 2 (in which the same set of practice and main trials was imposed for two days), and 45 trials in experiment 3.

We utilized a perturbation paradigm to investigate how the subjects modified their jumping motions when experiencing sensory prediction errors. For trials with a perturbation *p*, the position of the cursor was displayed at max ${k}_{s}+p$. The subjects needed to modify their jumping motions to achieve a lower (when $p > 0$) or higher (when $p < 0$) jump height. When the displayed jump height was close to the target height ($|d-({\rm{\max }}\,{k}_{s}+p)| < 0.02$), the subjects heard the coin-clattering sound, indicating that the jumping motion was successful.

### Task-relevant and task-irrelevant variabilities

Under the condition ${\boldsymbol{X}}={{\boldsymbol{X}}}_{{\rm{rel}}}+{{\boldsymbol{X}}}_{{\rm{irr}}}$ (see the *Results* section for details), the variance of the *i*th component of ***X***, denoted by *X*_*i*_, can be calculated as follows:

6 $$\begin{array}{rcl}{\rm{Var}}({X}_{i}) & = & \frac{1}{T}\,\sum _{t=1}^{T}\,{X}_{i,t}^{2}\\  & = & \frac{1}{T}\,\sum _{t=1}^{T}\,{({X}_{i,t}^{{\rm{rel}}}+{X}_{i,t}^{{\rm{irr}}})}^{2}\\  & = & {\rm{Var}}({X}_{i}^{{\rm{rel}}})+{\rm{Var}}({X}_{i}^{{\rm{irr}}})+2{\rm{Cov}}({X}_{i}^{{\rm{rel}}},{X}_{i}^{{\rm{irr}}}),\end{array}$$

where *X*_*i*,*t*_ is *X*_*i*_ in the *t*th trial, ${X}_{i,t}^{{\rm{rel}}}$ is the *i*th component of ***X***^rel^ in the *t*th trial, ${X}_{i,t}^{{\rm{irr}}}$ is the *i*th component of ***X***^irr^ in the *t*th trial, and ${\rm{Cov}}({X}_{i}^{{\rm{rel}}},{X}_{i}^{{\rm{irr}}})$ is the covariance between ${X}_{i}^{{\rm{rel}}}$ and ${X}_{i}^{{\rm{irr}}}$. Notably, in the current experimental setting, the values of ${\rm{Cov}}({X}_{i}^{{\rm{rel}}},{X}_{i}^{{\rm{irr}}})$ in the analyzed trials were close to 0. We thus considered only ${\rm{Var}}({X}_{i}^{{\rm{rel}}})$ and ${\rm{Var}}({X}_{i}^{{\rm{irr}}})$.

### Properties of ridge regression

Ridge regression showed high prediction power in the presence of measurement noise in ***d***. In a traditional regression analysis framework, in the presence of noise ***ξ*** with a mean of 0, the standard deviation is *σ*_0_, the covariance is 0, and the weight values ***w*** should be determined by minimizing

7 $${E}_{{\rm{regress}}}=\frac{1}{2}{({\boldsymbol{d}}+{\boldsymbol{\xi }}-{\boldsymbol{Xw}})}^{T}({\boldsymbol{d}}+{\boldsymbol{\xi }}-{\boldsymbol{Xw}}).$$

To enhance the fit quality and predictive power in the presence of noise, it is necessary to calculate ***w*** to maximize the power averaged across all possible noise patterns. The cost function averaged across all possible noise patterns can be written as

8 $$\langle {E}_{{\rm{regress}}}\rangle =\frac{1}{2}{({\boldsymbol{d}}-{\boldsymbol{Xw}})}^{T}({\boldsymbol{d}}-{\boldsymbol{Xw}})+\frac{{\sigma }_{o}^{2}}{2}{{\boldsymbol{w}}}^{T}{\boldsymbol{w}},$$

which is equivalent to the cost function considered in the ridge regression procedure (equation (1)) when ${\sigma }_{o}^{2}=\lambda$ (where *λ* is the regularization parameter for ridge regression). The equivalence between equations (1) and (8) indicates that ridge regression enables the selection of the best ***w*** to predict ***d*** in the presence of measurement noise while avoiding overfitting. This equivalence also suggests that the regularization parameter *λ* corresponds to the variance of the observation noise, ${\sigma }_{o}^{2}$.

Ridge regression enables the appropriate estimation of ***w*** based on the normalized ***d*** and ***X*** (i.e., the mean and standard deviation of ***d*** and ***X*** should be normalized to be 0 and 1, respectively): $\frac{1}{T}\,{\sum }_{t=1}^{T}\,{d}_{t}=0$, $\frac{1}{T}\,{\sum }_{t=1}^{T}\,{y}_{t}^{2}=1$, $\frac{1}{T}\,{\sum }_{t=1}^{T}\,{X}_{i,t}=0$, and $\frac{1}{T}\,{\sum }_{t=1}^{T}\,{X}_{i,t}^{2}=1$ ($d=1,\ldots ,D$). All the results in the current study were obtained based on such normalized data. Without normalization, *w*_*i*_ will be estimated to be large when *X*_*i*,*t*_ shows small fluctuations and vice versa, despite regularization with the parameter *λ* being imposed equally on all *w*_*i*_ values; therefore, normalization, especially in ***X***, is indispensable for estimating appropriate ***w*** values. Notably, the normalization did not affect the interpretation of the data in this study because it was possible to restore the original, unnormalized data by adding the original mean ${m}_{i}=\frac{1}{T}\,{\sum }_{t=1}\,{X}_{i,t}^{{\rm{original}}}$ and multiplying by the original standard deviation ${\sigma }_{i}=\sqrt{\frac{1}{T}\,{\sum }_{t=1}\,{({X}_{i,t}^{{\rm{original}}})}^{2}-{m}_{d}^{2}}$. To satisfy ${\sum }_{i=1}^{D}\,{w}_{i}{X}_{i,t}={\sum }_{i=1}^{D}\,{\tilde{w}}_{i}{X}_{i,t}^{{\rm{original}}}$, $\tilde{{\boldsymbol{w}}}$ should be divided by *σ*_*i*_ $({\tilde{w}}_{i}=\frac{{w}_{i}}{{\sigma }_{i}})$, and ${\sum }_{i=1}^{D}\,{\tilde{w}}_{i}{m}_{i}$ should then be subtracted, where ***w*** corresponds to the unnormalized data. In summary, while normalization was indispensable for appropriately estimating ***w***, it did not affect the results at all.

### Parabolic representation of the jump height, three candidate input data types, and the UCM and GEM methods

The vertical position of the marker attached to the subject’s back was used to determine the jump height in the current study. We expected that the jump height could be well predicted based on the position *p* and velocity *b* of the back marker at the time of release as follows:

9 $$h=p+\frac{{v}^{2}}{2g},$$

where $g\simeq 9.8\,m$/*s*^2^. In the joint angle representation, *p* and *v* are written as follows:

10 $$p=\sum _{i=1}^{4}\,{l}_{i}\,\sin \,{q}_{i}$$

and

11 $$v=\sum _{i=1}^{4}\,{l}_{i}{\dot{q}}_{i}\,\cos \,{q}_{i},$$

where *l*_*i*_ is the length of the *i*th limb segment (i.e., *l*_1_ is the length between the right toe and heel, *l*_2_ is the length between the right heel and knee, *l*_3_ is the length between the right knee and hip, and *l*_4_ is the length between the hip and back). When using the UCM method (blue crosses in Fig. 4B), we calculated the task-relevant and task-irrelevant variabilities based on equations (10) and (11).

Using equations (10) and (11), the predicted jump height *h* can be written as

12 $$h=\sum _{i=1}^{4}\,{l}_{i}\,\sin \,{q}_{i}+\frac{1}{2g}\,\sum _{i=1}^{4}\,\sum _{j=1}^{4}\,{l}_{i}{l}_{j}{\dot{q}}_{i}{\dot{q}}_{j}\,\cos \,{q}_{i}\,\cos \,{q}_{j}.$$

The first type of candidate input data for ridge regression consisted of the joint angles and angular velocities (blue line in Fig. 4A). The second candidate data type consisted of functions for the forward kinematics of the position and velocity of the hip joint (equations (10) and (11), red line in Fig. 4A). The third candidate data type consisted of the functions given in equation (12) (orange line in Fig. 4A). When using the GEM method (green crosses in Fig. 4B), we calculated the task-relevant and task-irrelevant variabilities based on equation (12).

### Relation between ridge regression and PCA

The relation between the ridge regression and PCA procedures can be analytically determined by decomposing ***X*** via SVD: ${\boldsymbol{X}}={\boldsymbol{UD}}{{\boldsymbol{V}}}^{T}$, where ${\boldsymbol{U}}\in {{\bf{R}}}^{T\times T}$ is an orthogonal matrix, ${\boldsymbol{D}}\in {{\bf{R}}}^{T\times D}$ includes the square root of the *i*th eigenvalue of ***X***^*T*^***X*** as each element (*i*, *i*) and ${D}_{i,j}=0$ when $i\ne j$, and ${\boldsymbol{V}}\in {{\bf{R}}}^{D\times D}$ is an orthogonal matrix. Using SVD and equation (2), the predicted output *h*_*t*_ can be written as follows:

13 $${h}_{t}={{\boldsymbol{X}}}_{t}{{\boldsymbol{w}}}^{\ast }={\boldsymbol{UD}}{({{\boldsymbol{D}}}^{T}{\boldsymbol{D}}+\lambda {\boldsymbol{I}})}^{-1}{{\boldsymbol{D}}}^{T}{{\boldsymbol{U}}}^{T}{\boldsymbol{d}}=\sum _{i=1}^{{\rm{\min }}(T,D)}\,\frac{{\lambda }_{i}^{2}}{{\lambda }_{i}^{2}+\lambda }{\rm{Corr}}({\boldsymbol{X}}{{\boldsymbol{v}}}_{i},{\boldsymbol{d}}){u}_{i,t},$$

where min(*T*, *D*) determines the rank of ***X***, ${\lambda }_{i}^{2}$ is an eigenvalue of ***X***^*T*^***X***, Corr(·, ·) denotes the correlation between two vectors, ***v***_*i*_ is the eigenvector of ***X***^*T*^***X*** corresponding to ${\lambda }_{i}^{2}$, and *u*_*i*,*t*_ is the (*i*, *t*) component of ***U***. On the other hand, PCA enables the decomposition of ***X***_*t*_ using

14 $${{\boldsymbol{X}}}_{t}=\sum _{i=1}^{{\rm{\min }}(T,D)}\,{\lambda }_{i}{u}_{i,t}{{\boldsymbol{v}}}_{i}.$$

This equation indicates that the motion data can be decomposed into eigenvectors (PCs) with weights of ${\lambda }_{i}{u}_{i,t}$. As seen by comparing equations (13) and (14), ridge regression enables the prediction of the output data by means of weighting based on the *i*th eigenvector with weight $\frac{{\lambda }_{i}^{2}}{{\lambda }_{i}^{2}+\lambda }{u}_{i,t}$ (notably, $\frac{{\lambda }_{i}^{2}}{{\lambda }_{i}^{2}+\lambda }$ is a monotonic function with respect to *λ*_*i*_). An important difference between PCA and ridge regression is whether the task relevance of the *i*th eigenvector, ${\rm{Corr}}({\boldsymbol{X}}{{\boldsymbol{v}}}_{i},{\boldsymbol{d}})$, is considered. Whereas PCA relies only on the eigenvalues, ridge regression considers both (the nonlinearly transformed) eigenvalues and the task relevance. Ridge regression can thus be considered an extended version of PCA that determines the relevance of each PC to the task.

For PCA, we find the relation between the explained variance and prediction power as follows: For an explained variance of *z*%, we determine the number of PCs based on ${n}_{z}={{\rm{\min }}}_{n}\,\frac{{\sum }_{i=1}^{n}\,{\lambda }_{i}^{2}}{{\sum }_{i=1}^{{\rm{\min }}(T,D)}\,{\lambda }_{i}^{2}} > \frac{z}{100}$ (i.e., the minimum number of PCs that collectively explain at least z% of the variance). Once *n*_*z*_ has been determined, the motion data can be reconstructed as ${\tilde{{\boldsymbol{X}}}}_{t}={\sum }_{i=1}^{{n}_{z}}\,{\lambda }_{i}{u}_{i,t}{{\boldsymbol{v}}}_{i}$. We then multiply ${\tilde{{\boldsymbol{X}}}}_{t}$ by ${\sum }_{i=1}^{{n}_{z}}\,{{\boldsymbol{v}}}_{i}^{T}$ from the right-hand side, resulting in ${\tilde{d}}_{t}={\tilde{{\boldsymbol{X}}}}_{t}\,{\sum }_{i=1}^{{n}_{z}}\,{{\boldsymbol{v}}}_{i}^{T}={\sum }_{i=1}^{{n}_{z}}\,{\lambda }_{i}{u}_{i,t}$. Finally, we calculate the correlation between the observed jump height *d*_*t*_ and ${\tilde{d}}_{t}$, as shown in Fig. 4D–F.

## Data Availability

The datasets analyzed in the current study are available from the corresponding author upon reasonable request.
